# Telemetric Assessment of Continuous Positive Airways Pressure (CPAP) Effectiveness and Adherence in Obstructive Sleep Apnea during COVID-19 Pandemic

**DOI:** 10.3390/biomedicines10051011

**Published:** 2022-04-27

**Authors:** Monika Michalek-Zrabkowska, Rafal Poreba, Pawel Gac, Weronika Frosztega, Anna Wojakowska, Mieszko Wieckiewicz, Justyna Kanclerska, Piotr Macek, Wlodzimierz Wieckiewicz, Grzegorz Mazur, Helena Martynowicz

**Affiliations:** 1 Department of Internal Medicine, Occupational Diseases, Hypertension and Clinical Oncology, Wroclaw Medical University, 213 Borowska St., 50-556 Wroclaw, Poland; monika.michalek1@gmail.com (M.M.-Z.); rafal.poreba@umw.edu.pl (R.P.); weronika.frosztega@gmail.com (W.F.); anna.wojakowska@umw.edu.pl (A.W.); kanclerska.justyna@gmail.com (J.K.); piotr.macek@student.umw.edu.pl (P.M.); grzegorz.mazur@umw.edu.pl (G.M.); helena.martynowicz@umw.edu.pl (H.M.); 2 Division of Environmental Health and Occupational Medicine, Department of Population Health, Wroclaw Medical University, 7 Mikulicza-Radeckiego St., 50-345 Wroclaw, Poland; 3 Department of Experimental Dentistry, Wroclaw Medical University, 26 Krakowska St., 50-425 Wroclaw, Poland; mieszko.wieckiewicz@umw.edu.pl; 4 Department of Prostodontics, Wroclaw Medical University, 26 Krakowska St., 50-425 Wroclaw, Poland; wlodzimierz.wieckiewicz@umw.edu.pl

**Keywords:** obstructive sleep apnea (OSA), COVID-19, SARS-CoV-2, continuous positive airways pressure, CPAP, adherence

## Abstract

Obstructive sleep apnea is the most common sleep-related breathing disorder. In the pandemic times of the new coronavirus SARS-CoV-2, CPAP (Continuous Positive Airway Pressure) therapy of obstructive sleep apnea became even more challenging. After the pandemic outbreak in March 2020, most CPAP treatment recommendations changed because of rising concerns about CPAP usage safety for patients and their families. Therefore, we examined the effectiveness of CPAP and adherence to the therapy of 149 adults with obstructive sleep apnea in the period of two years from 4 March 2019 to 3 March 2021 (before pandemic breakout and during the first year of pandemic). Data on CPAP parameters and adherence to therapy were obtained via a telemetric system. Together, our results demonstrated that the COVID-19 pandemic had no significant impact on CPAP therapy parameters and adherence in whole study group. However, detailed analysis acknowledged that some demographic and clinical features influenced CPAP therapy. The results showed that across subgroups of patients differentiated on the basis of age, gender, co-existing diabetes mellitus, or hypertension, the COVID-19 pandemic seemed to affect CPAP effectiveness. Our results provide a good starting point for discussion on CPAP therapy recommendations during pandemic times.

## 1. Introduction

Obstructive sleep apnea (OSA) is a common sleep disorder, connected with events of apnea or hypopnea as the result of the collapse of the upper airways during sleep [1]. Some signs and symptoms of OSA include loud snoring, episodes of apnea reported by “sleep witness”, excessive daytime sleepiness, sleep fragmentation, nocturia, and depression. According to Benjafield et al., almost 1 billion people are affected with obstructive sleep apnea and in some countries, the prevalence of OSA is over 50% of the population [2]. Obstructive sleep apnea is associated with multiple complications and consequences for general health as follows: hypertension, cardiac arrythmia, stroke, metabolic syndrome, diabetes mellitus, depression, and cognitive dysfunction [3]. CPAP (Continuous Positive Airway Pressure) is the gold standard therapy for OSA patients. It can be described as pneumatic support for tissues of the upper airway tract that stents the airways opened constantly during apnea or hypopnea events [4]. However, the usage of a CPAP device during COVID-19 pandemic became an emerging problem [5].

SARS-CoV-2 virus is a causative factor of COVID-19 disease, which was characterized by World Health Organization (WHO) as a pandemic in March 2020 [6]. Up to 21 March 2022, almost 469 million people worldwide have been infected with the coronavirus, and more than 6 million have died [7]. The number of COVID-19 cases is still rising rapidly, thus being a burden on the global health care system.

As has been previously reported in the literature, patients with chronic comorbidities as hypertension, cardiovascular system disease, diabetes, kidney failure, cerebrovascular disease, lung disease, obesity, male gender, and older age are especially exposed to the coronavirus disease complications [8,9,10,11]. The relationship between OSA and COVID-19 is still insufficiently determined; however, similar risk factors and comorbidities of both of them were emphasized in recent literature, for example, older age, male gender, obesity, cardiovascular diseases, arterial hypertension, and diabetes [5,12,13]. Moreover, the aforementioned conditions were considered probable determinants of poor COVID-19 outcomes. As was shown in the CORNADO study, treated obstructive sleep apnea in diabetic patients admitted to hospital due to coronavirus infection was independently associated with increased mortality at day 7 [14]. Although the pathophysiological mechanism of cardiovascular complications of coronavirus SARS-CoV-2 infection is still insufficiently investigated, data confirm that patients with co-existing COVID-19 and cardiovascular diseases are at increased risk of poor outcomes [15].

The usage of a CPAP device during pandemic became an emerging problem because of rising concerns of physicians and patients. The main issues involved patients’ and family members’ safety, difficulties in CPAP tolerance because of fever, cough, and other symptoms of viral infection, and low adherence to therapy. The problem of increased aerosol production and potential viral spread into the local environment while using a CPAP was also raised [5,16,17]. AASM recommendations constitute that the decision whether continue or cease CPAP treatment during coronavirus infection should be made according to individual’s risk assessment [18].

For this study, it was of interest to explore how the COVID-19 pandemic has influenced usage and effectiveness of the CPAP therapy across demographic and clinical subgroups of patients.

## 2. Materials and Methods

The study group was selected from the patients of the Sleep Laboratory in the Department of Internal Medicine, Occupational Diseases, Hypertension, and Clinical Oncology at Wroclaw Medical University in Poland. The data were obtained from a cloud database of CPAP users via the telemetric patient management system.

We examined the adherence to the therapy in the period of two years from 4 March 2019 to 3 March 2021. Two similar periods of time were compared, 365 days each. Inasmuch as the first positive case of coronavirus infection in Poland was reported on 4 March 2020, the effectiveness of CPAP treatment was compared initially 1 year before COVID-19 pandemic outbreak (first period) and 1 year after (second period). This was a long-term observational study of patients diagnosed with OSA during CPAP therapy.

### Participants

A total number of *n* = 1159 patients were identified through the telemetric system database. Finally, after removing records from in-patient and out-patient CPAP trials, incomplete records, and data from patients who dismissed CPAP treatment, the study group consisted of *n* = 149 adult individuals, *n* = 109 (73.15%) male and *n* = 40 (26.85%) female with diagnosed obstructive sleep apnea who had moderate to severe obstructive sleep apnea. The mean age of the participants was 62.26 ± 10.82 years; n = 18 had coronary artery disease, *n* = 36 had diabetes mellitus, *n* = 104 were diagnosed with arterial hypertension, *n* = 95 were obese (BMI ≥ 30 kg/m^2^), *n* = 8 had asthma. The inclusion criteria consisted of age of participant above 18 years old, apnea-hypopnea index (AHI) ≥ 15 (moderate to severe OSA), and wirelessly monitored CPAP therapy at home. The exclusion criteria were as follows: (1) diagnosed central apnea, (2) severe respiratory failure, (3) severe heart failure, or (4) active neoplastic process.

During pandemic outbreak, all patients continued their CPAP therapy at home, supervised by a physician with the help of the telemetric healthcare system. Three types of machines and setups were used: CPAP with one continuous pressure level of air delivered, automatic positive airway pressure (APAP) with automatically adjusted air pressure accordingly to severity of respiratory event, and Auto CPAP for women designed to manage female characteristics of OSA, for example respiratory cluster events in REM (rapid eye movement) sleep.

Informed and written consent was obtained from each patient. The study protocol was approved by the Ethic Committee of the Wroclaw Medical University (the Consent No. KB 308/2018).

Statistical analyses were performed using the Dell Statistica 13.1 application (Dell Inc., Round Rock, TX, USA). For quantitative variables, arithmetic means and standard deviations were calculated for qualitative variables-percentages. The distribution of variables was checked with the Shapiro–Wilk test. The hypotheses were tested with *t*-tests dedicated to unrelated and related variables, respectively. The differences between the mean values at *p* value < 0.05 were considered statistically significant.

## 3. Results

The average AHI of the study group at the beginning of study (treatment) was 46.54 ± 23.11 events/hour of sleep, the mean oxygen desaturation index (ODI) was 43.08 ± 23.24 events/hour, the average saturation was 90.00 ± 8.29%, the minimal saturation estimated 75.09 ± 11.74%, and duration of saturation below 90% was 25.18 ± 23.45% of time.

Compliance and CPAP therapy characteristics of whole study group before and after pandemic outbreak are demonstrated in Table 1.

According to the gender of study participants, mean AHI and ODI indexes were estimated to be, respectively, 47.89 ± 23.18 and 44.43 ± 24.00 in men; 42.99 ± 22.84 and 39.49 ± 20.95 in women at the beginning of CPAP treatment. Mean age was 60.79 ± 11.36 years in men and 67.15 ± 7.35 years in women (*p* < 0.001). Differences in CPAP therapy supplied by the telemetric system between male and female individuals was statistically significant in terms of average maximum CPAP pressure (11.58 ± 3.05 cm H_2_O in males and 14.33 ± 3.08 cm H_2_O in females; *p* < 0.01 before pandemic breakout vs. 11.66 ± 3.09 cm H_2_O in males and 13.83 ± 3.59 cmH_2_O in females, *p* < 0.05 after pandemic breakout). Average HI estimated 0.82 ± 1.03 events/hour in men and 0.41 ± 0.34 events/hour in women (*p* < 0.05) before pandemic breakout vs. 0.90 ± 1.51 events/hour and 0.35 ± 0.31 events/hour (*p* < 0.05), respectively, after pandemic breakout; Table 2).

According to the age of participants, Table 3 shows mean CPAP parameters and average usage time in subjects below or equal to 65 years old and more than 65 years. Before CPAP therapy, mean AHI and ODI indexes were estimated to be, respectively, 49.03 ± 25.06 and 44.83 ± 25.46 in the age group below or equal to 65 years; 43.30 ± 20.04 and 40.81 ± 19.95, respectively, in the age group above 65 years old. Mean oxygen saturation of arterial blood at the beginning of the treatment was accordingly 89.29% ± 10.83 in the study group below or equal to 65 years old and 90.91% ± 2.45 in the study group over 65 years. The differences between age groups in terms of polysomnographic parameters at the beginning of CPAP treatment were insignificant. Statistically significant differences in CPAP therapy parameters and compliance between these age groups were bolded in Table 3.

In subgroups of normal-to-overweight (BMI < 30 kg/m^2^) and obese (BMI ≥ 30 kg/m^2^) individuals polysomnographic parameters varied significantly in terms of AHI, ODI, and duration of oxygen saturation below 90% at the beginning of study period. Before CPAP therapy, mean AHI was estimated to be 37.49 ± 17.21 events/hour in patients with BMI < 30 kg/m^2^ and 51.96 ± 24.46 events/hour in obese subgroup of patients (*p* < 0.001). Mean ODI indexes were estimated to be 32.43 ± 17.79 events/hour in normal-to-overweight patients and 49.13 ± 23.70 events/hour in obese individuals (*p* < 0.001). Mean oxygen saturation of arterial blood at the beginning of treatment was accordingly 89.77% ± 13.57 in the study group with BMI < 30 kg/m^2^ and 90.08% ± 3.17 in the study group with BMI ≥ 30 kg/m^2^. Duration of saturation below 90% in normal-to-overweight patients estimated 16.53% ± 19.21 whereas in obese patients, it was 30.37% ± 24.24 (*p* < 0.001). Parameters of CPAP therapy of both subgroups are involved in Table 4.

Table 5, Table 6 and Table 7 show differences in CPAP parameters and compliance to therapy between compared periods of time in individuals with comorbidities and without it: hypertension (Table 5), diabetes (Table 6), and asthma (Table 7).

The difference between subgroups with and without hypertension was statistically significant in terms of age-mean age of normotensive group estimated 57.12 ± 12.31 years whereas in the hypertensive group, it was 64.32 ± 9.57 years (*p* < 0.001). Average polysomnographic parameters at the beginning of CPAP therapy were estimated as follows: average AHI and ODI 43.45 ± 23.98 events/hour and 39.01 ± 24.05 events/hour, respectively, in normotensives; 48.04 ± 22.86 events/hour and 44.92 ± 22.85 events/hour in hypertensives. Mean oxygen saturation of arterial blood at the beginning of treatment was accordingly 88.38% ± 15.42 in normotensive study group and 90.55% ± 2.62 in hypertensive study group (Table 5).

The age difference between subgroups with and without diabetes mellitus was statistically significant. The average age of the non-diabetic group was 61.20 ± 11.45 years, whereas in the diabetic subgroup it was 65.61 ± 8.37 years (*p* < 0.05). Polysomnographic parameters before CPAP treatment indicated: AHI and ODI 48.08 ± 23.15 and 43.97 ± 23.14, respectively, in non-diabetics; 41.64 ± 22.43 and 40.18 ± 23.18 in diabetics. Average oxygen saturation of arterial blood was accordingly 89.72% ± 9.55 in non-diabetics and 90.80% ± 2.50 in diabetics.

Before pandemic breakout, average median air leakage estimated 4.57 ± 6.54 L/min in non-diabetics and 7.53 ± 8.59 L/min in diabetics (*p* < 0.05). During the first year of the pandemic, the mean median leakage was 5.38 ± 7.54 L/min in patients without diabetes and 10.15 ± 12.42 L/min in diabetic individuals (*p* < 0.01). Moreover, statistically relevant differences between non-diabetic and diabetic individuals during the pandemic period were found in parameters of average air leaks 95th percentile (19.89 ± 14.26 L/min vs. 27.43 ± 19.57 L/min; *p* < 0.01) and average maximum air leaks (30.71 ± 16.28 L/min vs. 39.69 ± 19.30 L/min, *p* < 0.01). Statistically significant differences between non-diabetic and diabetic individuals are bolded in Table 6.

Table 7 show differences in CPAP therapy parameters between individuals with and without asthma. No statistical differences were found between both groups in average polysomnographic parameters before CPAP therapy. Mean age in the non-asthmatic group was 62.12 ± 10.69 years, 65.75 ± 14.14 years in individuals with asthma. AHI and ODI indexes were estimated to be, respectively, 47.21 ± 23.00 events/hour and 43.72 ± 23.09 events/hour in non-asthmatics; 38.00 ± 24.81 events/hour and 36.29 ± 24.85 events/hour, respectively, in asthmatics. Mean oxygen saturation of arterial blood was estimated to be, respectively, 89.92% ± 8.60 in individuals without asthma and 90.69% ± 1.95 in patients with co-existing asthma (Table 7).

## 4. Discussion

The results of this study show how demographic and clinical factors have influenced usage and effectiveness of the CPAP therapy during the COVID-19 pandemic. Together, the present findings demonstrated that the COVID-19 pandemic had no significant impact on CPAP therapy in the whole study group. However, detailed comparisons between demographic and clinical subgroups confirmed the influence of COVID-19 pandemic on OBS treatment with CPAP. The statistically significant impact on CPAP parameters was showed in patients with co-morbidities: diabetes mellitus and hypertension. Moreover, demographic factors such as age and gender also influenced CPAP therapy parameters and adherence before the pandemic outbreak and during the first year of pandemic time.

The long-term adherence to CPAP therapy of obstructive sleep apnea was also a challenging problem before the pandemic outbreak [19,20]. There are many factors influencing CPAP adherence, e.g., usage comfort, costs of purchase and maintenance of PAP device, psychosocial factors, and cognitive ability [21,22,23]. Although data on CPAP adherence during COVID-19 pandemic are limited, overall findings suggest that CPAP therapy adherence increased [24,25]. Our results indicate that average median usage in individuals above 65 years old before and after the pandemic outbreak was significantly increased compared with the age group below or equal to 65 years. These findings are in line with assumptions that older patients, who are at increased risk of poor coronavirus infection outcomes, are more adherent. However, in contrast to our findings, Demirovic et al. demonstrated that responders younger than 58 years had increased adherence to CPAP treatment during the lockdown period [25]. One concern about the aforementioned study was that the analyzed lockdown period was 40 days. It is important to note that the present evidence relies on one-year periods before pandemic outbreak and during the first year of the pandemic, to obtain the most robust results. The fact that CPAP adherence in older age group was increased despite statistically higher mean maximum air leaks before the pandemic outbreak highlights the treatment motivation in the older age group. This may demonstrate that the potential benefits from OSA therapy for overall health were key stimuli to CPAP use and were more important in subjects over 65 years old than the inconvenience related with air leakage. The difference was not statistically significant after the COVID-19 pandemic. According to the apnea-hypopnea index in both age groups, average AHI was significantly increased before the pandemic outbreak in the older age group. The difference in AHI between those age groups was insignificant during the first year of the pandemic. This appears to be an effect of better therapy adherence. As discussed by Attias et al., patients’ perception of both OSA and COVID-19-related risk influenced CPAP adherence and improved it [24]. Although initial recommendations on CPAP usage safety were inconclusive [5], more up-to-date protocols underline the importance of OSA diagnosing and treatment in consonance with healthcare providers’ instructions and restrictive hygienic routine [26].

According to the second demographic subgroup of patients, differentiated based on gender, findings showed significantly increased average maximum CPAP pressure in women compared with men both before and after the pandemic outbreak. A further finding is that the hypopnea index was significantly increased in male subjects both before and after the pandemic outbreak. It is difficult to explain such results within the context of age. The mean age of the female group was 67.15 years whereas the mean age of the male group was 60.47 years. The difference was statistically significant. Pre-pandemic studies emphasized the role of age and gender differences in the OSA diagnosing process [27,28] and treatment [28,29,30]. A recent meta-analysis of 57 studies with 221,195 participants by Abate et al. demonstrated that the symptomatic COVID-19 prevalence was found to be higher in the male gender than in the female [31]. The study was predominantly focused on the Chinese population which is considered the main limitation of this report.

Overall, gender-related demographic aspects of research suggest that the COVID-19 pandemic outbreak slightly affected CPAP therapy parameters in both genders; however, these assumptions should be addressed in future studies due to the influence of coronavirus on sex groups.

Our findings on CPAP therapy in participants with co-morbidities at least hint that in hypertensive and diabetic OSA patients, there were statistically significant differences between CPAP parameters before the pandemic outbreak and during the first year of the pandemic.

We showed that the hypertensive subgroup of patients had a significantly higher mean CPAP set pressure independently from the study period. Although several studies suggested that hypertensive individuals are at increased risk of severe OSA and may need intensive CPAP treatment [32,33,34], the influence of the coronavirus SARS-CoV-2 pandemic on CPAP parameters in OSA treatment is still insufficiently explored. To our knowledge, no prior studies have examined this relationship directly. A still unsolved question is whether COVID-19 influences CPAP therapy outcomes and effectiveness. As far as we know, hypertension is strongly associated with obstructive sleep apnea [35]. Furthermore, untreated OSA could lead to development of resistant hypertension. Miller et al. concluded that many of the risk factors for OSA are also risk factors for poor coronavirus infection outcomes [12]. Thus, hypertension management in OSA patients with/or without coexisting COVID-19 still plays an important role in the reduction of OSA symptoms.

According to the diabetic subgroup, we observed a tendency to increased leakage during the first year of the pandemic, with a relevant increase in diabetics compared to non-diabetic individuals. The results lead to the conclusion that during the coronavirus pandemic, improvement of CPAP therapy, especially precise mask fitting and CPAP intolerance management could prove quite beneficial to patients [26,36]. Recent research by Demirovic et al. demonstrated that CPAP adherence during lockdown in subjects with diabetes mellitus increased [24]. However, a closer look at the literature on obstructive sleep apnea CPAP therapy in the pandemic era reveals a number of gaps and shortcomings. To our knowledge, observational studies on CPAP therapy and its parameters in the COVID-19 era remain unpublished. Broadly translated, our findings indicate that healthcare providers and sleep laboratory physicians should focus on successfully conducting CPAP treatment. Behavioral interventions, implementation of telemedicine, and telemetric supervision of CPAP therapy and adherence are currently accepted and recommended methods of OSA management during the COVID-19 pandemic [16,24,37,38,39].

The apparent limitations of the present study naturally include lack of gender parity in the study group and very limited available literature on this topic. It is important to note that the present evidence relies on early pandemic data. Another limitation involves the issue of single center data presented in our study. During early pandemic times, access to inpatient healthcare was limited due to a high risk of infection and lack of safety procedures, thus multicenter comparisons were impossible. According to potential selection biases, the study group was representative for patients with moderate and severe obstructive sleep apnea population in terms of age, weight, height, BMI, co-morbidities, and CPAP therapy inclusion criteria. After removing data on CPAP usage which did not meet inclusion and exclusion criteria and incomplete records, we included the remaining CPAP therapy records (*n* = 149). However, information about selection biases associated with the presented data is limited to our Sleep Laboratory.

Overall, our results demonstrated the influence of demographic and clinical factors across subgroups of patients on CPAP effectiveness in obstructive sleep apnea during the COVID-19 pandemic. The age and gender of the studied population significantly influenced CPAP therapy parameters and effectiveness during the pandemic outbreak. Statistically significant differences in CPAP usage parameters were also found in diabetic and hypertensive subgroups of study population.

Current research can only be considered a first step towards a more profound understanding of CPAP therapy goals and challenges in the pandemic era.

## 5. Conclusions

Collectively, our results suggest that the COVID-19 pandemic had no significant impact on CPAP therapy in the whole study group. However, thorough analysis across subgroups of patients indicated that the demographic factors of age and gender had a notable impact on CPAP effectiveness in obstructive sleep apnea during the COVID-19 pandemic. Moreover, a detailed report on CPAP therapy showed that the first year of the pandemic slightly influenced CPAP use in diabetic and hypertensive subgroups of patients compared with individuals without co-morbidities. Future research should further investigate the association between the COVID-19 pandemic and CPAP treatment of obstructive sleep apnea and develop recommendations for patients with obstructive sleep apnea and increased risk of COVID-19 infection. In addition, these results warrant further management of CPAP therapy in OSA patients via a telemetric system during pandemic times.

## Figures and Tables

**Table 1:** CPAP therapy parameters and therapy adherence of study group before pandemic outbreak and during first year of pandemic time.

Parameter	Before Pandemic Outbreak *n* = 149	During Pandemic Time *n* = 149	*p*
Average usage days (% of year)	70.00 ± 32.45	69.74 ± 35.97	ns
Average usage ≥ 4 h/day (% of year)	63.63 ± 33.99	64.68 ± 36.99	ns
Average usage time (total days, min)	276.56 ± 154.43	291.01 ± 176.69	ns
Average usage time (usage days, min)	374.41 ± 102.63	373.83 ± 127.36	ns
Average set pressure (cm H_2_O)	9.55 ± 2.07	9.71 ± 2.12	ns
Average minimal pressure (cm H_2_O)	5.14 ± 1.63	5.13 ± 1.66	ns
Average maximum pressure (cm H_2_O)	12.25 ± 3.25	12.21 ± 3.32	ns
Average median air leaks (L/min)	5.24 ± 7.12	6.46 ± 9.06	ns
Average AI (*n*/hour)	1.89 ± 2.35	2.59 ± 6.08	ns
Average HI (*n*/hour)	0.71 ± 0.91	0.74 ± 1.30	ns
Average AHI (*n*/hour)	2.86 ± 4.32	3.34 ± 6.79	ns
Central apnea index (*n*/hour)	0.62 ± 1.32	0.66 ± 1.54	ns
Obstructive apnea index (*n*/hour)	1.09 ± 1.31	1.70 ± 5.55	ns

AI, apnea index; HI, hypopnea index; AHI, apnea-hypopnea index.

**Table 2:** CPAP therapy parameters and therapy adherence of male and female study participants before pandemic outbreak and during first year of pandemic time.

	Before Pandemic Outbreak			During Pandemic Time		
Parameter	Men (*n* = 109)	Women (n = 40)	*p*	Men (*n* = 109)	Women (*n* = 40)	*p*
Average usage days (% of year) ± SD	68.22 ± 32.40	74.85 ± 32.50	ns	69.68 ± 35.14	69.90 ± 38.40	ns
Avg. usage ≥ 4 h/day (% of year) ± SD	61.96 ± 34.23	68.15 ± 33.33	ns	64.72 ± 36.34	64.59 ± 39.02	ns
Average usage time (total days, min) ± SD	270.38 ± 156.90	293.36 ± 148.18	ns	294.54 ± 178.16	282.33 ± 175.02	ns
Average usage time (usage days, min) ± SD	373.74 ± 112.15	376.23 ± 71.79	ns	378.91 ± 132.95	361.31 ± 113.13	ns
Average set pressure (cm H_2_O) ± SD	9.67 ± 1.97	9.27 ± 2.34	ns	9.82 ± 1.97	9.44 ± 2.45	ns
Average minimal pressure (cm H_2_O) ± SD	5.08 ± 1.66	5.33 ± 1.61	ns	5.06 ± 1.70	5.33 ± 1.61	ns
Average maximum pressure (cm H_2_O) ± SD	**11.58 ± 3.05**	**14.33 ± 3.08**	**<0.01**	**11.66 ± 3.09**	**13.83 ± 3.59**	**<0.05**
Average median air leaks (L/min) ± SD	5.40 ± 7.49	4.82 ± 6.09	ns	6.63 ± 8.45	6.05 ± 10.54	ns
Average AI (*n*/hour) ± SD	1.92 ± 2.51	1.83 ± 1.86	ns	2.90 ± 7.06	1.84 ± 2.26	ns
Average HI (*n/*hour) ± SD	**0.82 ± 1.03**	**0.41 ± 0.34**	**<0.05**	**0.90 ± 1.51**	**0.35 ± 0.31**	**<0.05**
Average AHI (*n/*hour) ± SD	3.09 ± 4.90	2.24 ± 2.01	ns	3.80 ± 7.88	2.19 ± 2.40	ns
Central apnea index (*n/*hour) ± SD	0.65 ± 1.37	0.56 ± 1.17	ns	0.71 ± 1.67	0.53 ± 1.16	ns
Obstructive apnea index (*n*/hour) ± SD	1.08 ± 1.39	1.12 ± 1.08	ns	1.94 ± 6.52	1.11 ± 1.38	ns

SD, standard deviation; Avg., average; AI, apnea index; HI, hypopnea index; AHI, apnea-hypopnea index.

**Table 3:** CPAP therapy parameters and therapy adherence of study participants before pandemic outbreak and during first year of pandemic time differentiated on the basis of age.

	Before Pandemic Outbreak			During Pandemic Time		
Parameter	Age ≤ 65 y (*n* = 77)	Age > 65 y (*n* = 72)	*p*	Age ≤ 65 y (*n* = 77)	Age > 65 y (*n* = 72)	*p*
Average usage days (% of year) ± SD	67.73 ± 34.57	72.95 ± 29.47	ns	68.29 ± 36.17	71.55 ± 35.93	ns
Avg. usage ≥ 4 h/day (% of year) ± SD	61.89 ± 35.25	65.89 ± 32.41	ns	62.65 ± 36.65	67.21 ± 37.56	ns
Average usage time (total days, min) ± SD	263.71 ± 157.58	293.25 ± 149.83	ns	277.43 ± 173.10	308.00 ± 181.11	ns
Average usage time (usage days, min) ± SD	362.72 ± 108.52	389.62 ± 93.07	ns	360.64 ± 131.20	390.30 ± 121.51	ns
Avg. median usage (usage days, min) ± SD	**364.83 ± 100.43**	**398.24 ± 94.50**	**<0.05**	**362.35 ± 129.97**	**403.73 ± 139.13**	**<0.05**
Average set pressure (cm H_2_O) ± SD	9.73 ± 2.23	9.35 ± 1.88	ns	**10.18 ± 2.29**	**9.18 ± 1.78**	**<0.05**
Average minimal pressure (cm H_2_O) ± SD	5.33 ± 1.32	4.84 ± 2.03	ns	5.32 ± 1.36	4.84 ± 2.03	ns
Average maximum pressure (cm H_2_O) ± SD	12.68 ± 2.62	11.58 ± 4.05	ns	12.86 ± 2.58	11.26 ± 4.08	ns
Average median leaks (L/min) ± SD	4.94 ± 7.41	5.64 ± 6.77	ns	6.36 ± 9.13	6.60 ± 9.06	ns
Average maximum air leaks (L/min) ± SD	**27.34 ± 17.68**	**34.18 ± 16.19**	**<0.01**	30.92 ± 18.31	35.20 ± 15.66	ns
Average AI (*n*/hour) ± SD	**1.50 ± 1.53**	**2.40 ± 3.05**	**<0.05**	**1.79 ± 3.81**	**3.59 ± 8.00**	**<0.05**
Average HI (*n*/hour) ± SD	0.75 ± 1.03	0.65 ± 0.75	ns	0.87 ± 1.67	0.58 ± 0.56	ns
Average AHI (*n*/hour) ± SD	**2.25 ± 2.33**	**3.65 ± 5.94**	**<0.05**	2.67 ± 5.29	4.17 ± 8.27	ns
Central apnea index (*n*/hour) ± SD	**0.39 ± 0.69**	**0.92 ± 1.80**	**<0.01**	**0.43 ± 1.28**	**0.94 ± 1.79**	**<0.05**
Obstructive apnea index (*n*/hour) ± SD	0.95 ± 1.07	1.27 ± 1.56	ns	1.12 ± 3.47	2.42 ± 7.34	ns

SD, standard deviation; Avg., average; AI, apnea index; HI, hypopnea index; AHI, apnea-hypopnea index.

**Table 4:** CPAP therapy parameters and therapy adherence of study participants before pandemic outbreak and during first year of pandemic time differentiated on the basis of BMI.

	Before Pandemic Outbreak			During Pandemic Time		
Parameter	BMI < 30 (*n* = 52)	BMI ≥ 30 (*n* = 95)	*p*	BMI < 30 (*n* = 52)	BMI ≥ 30 (*n* = 95)	*p*
Avg. usage days (% of year) ± SD	66.04 ± 31.68	72.24 ± 32.93	ns	65.96 ± 36.90	71.97 ± 35.86	ns
Avg. usage ≥ 4 h/day (% of year) ± SD	60.14 ± 32.73	65.73 ± 34.85	ns	60.87 ± 37.61	67.03 ± 37.04	ns
Avg. usage time (total days, min) ± SD	257.10 ± 144.29	288.29 ± 160.32	ns	262.84 ± 165.68	307.45 ± 182.57	ns
Average usage time (usage days, min) ± SD	361.46 ± 105.86	381.98 ± 101.67	ns	366.49 ± 137.27	377.78 ± 123.53	ns
Average set pressure (cm H_2_O) ± SD	9.13 ± 1.73	9.75 ± 2.22	ns	9.15 ± 1.85	9.95 ± 2.21	ns
Average minimal pressure (cm H_2_O) ± SD	4.74 ± 1.33	5.41 ± 1.80	ns	4.72 ± 1.36	5.39 ± 1.83	ns
Avg. maximum pressure (cm H_2_O) ± SD	12.23 ± 3.18	12.28 ± 3.41	ns	12.11 ± 3.27	12.29 ± 3.47	ns
Average median air leaks (L/min) ± SD	5.44 ± 7.35	5.21 ± 7.10	ns	6.03 ± 7.57	6.79 ± 9.84	ns
Average AI (*n*/hour) ± SD	2.21 ± 2.21	1.74 ± 2.44	ns	2.88 ± 4.96	2.49 ± 6.67	ns
Average HI (*n*/hour) ± SD	0.65 ± 0.60	0.74 ± 1.05	ns	0.89 ± 1.83	0.67 ± 0.95	ns
Average AHI (*n*/hour) ± SD	2.86 ± 2.54	2.88 ± 5.08	ns	3.77 ± 6.56	3.16 ± 7.01	ns
Central apnea index (*n*/hour) ± SD	0.76 ± 1.01	0.56 ± 1.47	ns	0.74 ± 1.64	0.63 ± 1.51	ns
Obstructive apnea index (*n*/hour) ± SD	1.23 ± 1.53	1.02 ± 1.19	ns	1.89 ± 4.56	1.63 ± 6.07	ns

BMI, body mass index (kg/m^2^); SD, standard deviation; Avg., average; AI, apnea index; HI, hypopnea index; AHI, apnea-hypopnea index.

**Table 5:** CPAP therapy parameters and therapy adherence of hypertensive and non-hypertensive study participants before pandemic outbreak during first year of pandemic time.

	Before Pandemic Outbreak			During Pandemic Time		
Parameter	Non-Hypertensives (*n* = 42)	Hypertensives (*n* = 104)	*p*	Non-Hypertensives (*n* = 42)	Hypertensives (*n* = 104)	*p*
Average usage days (% of year) ± SD	69.93 ± 33.15	70.56 ± 32.30	ns	71.03 ± 32.52	69.97 ± 37.63	ns
Avg. usage ≥ 4 h/day (% of year) ± SD	63.98 ± 34.52	64.31 ± 33.72	ns	65.47 ± 34.68	65.41 ± 37.97	ns
Avg. usage time (total days, min) ± SD	270.51 ± 149.99	282.44 ± 156.84	ns	280.11 ± 151.75	300.09 ± 186.53	ns
Avg. usage time (usage days, min) ± SD	373.73 ± 109.25	377.09 ± 100.05	ns	379.34 ± 120.24	374.44 ± 129.62	ns
Average set pressure (cm H_2_O) ± SD	**8.73 ± 1.59**	**9.88 ± 2.16**	**<0.05**	**8.87 ± 1.60**	**10.05 ± 2.21**	**<0.05**
Avg. minimal pressure (cm H_2_O) ± SD	5.38 ± 1.15	5.03 ± 1.86	ns	5.40 ± 1.18	5.00 ± 1.88	ns
Avg. maximum pressure (cm H_2_O) ± SD	12.96 ± 3.13	11.91 ± 3.35	ns	13.27 ± 2.99	11.71 ± 3.46	ns
Average median air leaks (L/min) ± SD	3.87 ± 5.48	5.90 ± 7.71	ns	5.17 ± 7.59	7.14 ± 9.67	ns
Avg. air leaks 94th percentile (L/min) ± SD	**16.13 ± 12.79**	**21.22 ± 15.83**	**<0.05**	18.90 ± 15.09	23.06 ± 16.18	ns
Average AI *(n*/hour) ± SD	1.54 ± 1.83	2.06 ± 2.55	ns	2.17 ± 4.99	2.82 ± 6.57	ns
Average HI (*n*/hour) ± SD	0.59 ± 0.55	0.76 ± 1.04	ns	0.84 ± 1.89	0.71 ± 1.01	ns
Average AHI (*n*/hour) ± SD	2.14 ± 2.14	3.19 ± 4.97	ns	3.02 ± 6.79	3.54 ± 6.92	ns
Central apnea index (*n*/hour) ± SD	0.47 ± 0.75	0.69 ± 1.50	ns	0.44 ± 0.73	0.76 ± 1.78	ns
Obstructive apnea index (*n*/hour) ± SD	0.96 ± 1.40	1.16 ± 1.29	ns	1.57 ± 4.90	1.79 ± 5.90	ns

SD, standard deviation; Avg., average; AI, apnea index; HI, hypopnea index; AHI, apnea-hypopnea index.

**Table 6:** CPAP therapy parameters and therapy adherence of diabetic and non-diabetic study participants before pandemic outbreak during first year of pandemic time.

	Before Pandemic Outbreak			During Pandemic Time		
Parameter	Non-Diabetic (*n* = 110)	Diabetic (*n* = 36)	*p*	Non-Diabetic (*n* = 110)	Diabetic (*n* = 36)	*p*
Average usage days (% of year) ± SD	72.78 ± 31.55	63.37 ± 34.01	ns	73.06 ± 34.20	62.34 ± 39.90	ns
Avg. usage ≥ 4 h/day (% of year) ± SD	66.65 ± 32.51	56.54 ± 37.39	ns	67.82 ± 34.93	58.00 ± 42.25	ns
Average usage time (total days, min) ± SD	284.80 ± 146.37	261.49 ± 177.76	ns	300.07 ± 167.06	277.41 ± 205.20	ns
Avg. usage time (usage days, min) ± SD	374.92 ± 96.12	377.31 ± 124.18	ns	380.70 ± 113.13	360.47 ± 163.08	ns
Average set pressure (cm H_2_O) ± SD	9.64 ± 1.99	9.27 ± 2.27	ns	9.63 ± 2.09	9.78 ± 2.24	ns
Average minimal pressure (cm H_2_O) ± SD	5.19 ± 1.71	4.80 ± 1.10	ns	5.17 ± 1.75	4.80 ± 1.10	ns
Avg. maximum pressure (cm H_2_O) ± SD	12.17 ± 3.35	13.00 ± 2.92	ns	12.12 ± 3.43	13.00 ± 2.92	ns
Average median air leaks (L/min) ± SD	**4.57 ± 6.54**	**7.53 ± 8.59**	**<0.05**	**5.38 ± 7.54**	**10.15 ± 12.42**	**<0.01**
Avg. air leaks 95th percentile (L/min) ± SD	18.59 ± 13.93	22.90 ± 18.24	ns	**19.89 ± 14.26**	**27.43 ± 19.57**	**<0.01**
Average maximum air leaks (L/min) ± SD	29.09 ± 16.67	34.46 ± 19.36	ns	**30.71 ± 16.28**	**39.69 ± 19.30**	**<0.01**
Average AI *(n*/hour) ± SD	1.98 ± 2.48	1.90 ± 2.37	ns	2.35 ± 4.35	3.48 ± 9.91	ns
Average HI *(n*/hour) ± SD	0.67 ± 0.67	0.71 ± 0.92	ns	0.77 ± 1.33	0.70 ± 1.28	ns
Average AHI *(n*/hour) ± SD	3.00 ± 4.71	2.50 ± 3.14	ns	3.12 ± 5.33	4.19 ± 10.37	ns
Central apnea index (*n*/hour) ± SD	0.66 ± 1.45	0.52 ± 0.88	ns	0.65 ± 1.52	0.69 ± 1.68	ns
Obstructive apnea index *(n*/hour) ± SD	1.13 ± 1.38	0.96 ± 1.13	ns	1.48 ± 3.53	2.48 ± 9.61	ns

SD, standard deviation; Avg., average; AI, apnea index; HI, hypopnea index; AHI, apnea-hypopnea index.

**Table 7:** CPAP therapy parameters and therapy adherence of asthmatic and non-asthmatic study participants before pandemic outbreak and during first year of pandemic time.

	Before Pandemic Outbreak			During Pandemic Time		
Parameter	Non-Asthmatic (*n* = 139)	Asthmatic (*n* = 8)	*p*	Non-Asthmatic (*n* = 139)	Asthmatic (*n* = 8)	*p*
Average usage days (% of year) ± SD	69.28 ± 32.79	83.38 ± 25.91	ns	69.17 ± 36.40	81.88 ± 32.47	ns
Avg. usage ≥ 4 h/day (% of year) ± SD	62.80 ± 34.35	80.25 ± 26.41	ns	64.06 ± 37.47	78.88 ± 31.62	ns
Avg. usage time (total days, min) ± SD	272.56 ± 155.17	358.75 ± 138.20	ns	288.57 ± 179.44	351.62 ± 143.66	ns
Avg. usage time (usage days, min) ± SD	372.19 ± 103.56	418.88 ± 92.84	ns	371.14 ± 129.99	418.00 ± 83.30	ns
Average set pressure (cm H_2_O) ± SD	9.57 ± 2.09	9.20 ± 2.17	ns	9.73 ± 2.13	9.20 ± 2.17	ns
Average minimal pressure (cm H_2_O) ± SD	5.18 ± 1.68	4.67 ± 1.15	ns	5.16 ± 1.72	4.67 ± 1.15	ns
Avg. maximum pressure (cm H_2_O) ± SD	12.14 ± 3.34	14.00 ± 2.00	ns	12.23 ± 3.37	12.00 ± 4.00	ns
Average median air leaks (L/min) ± SD	2.30 ± 7.19	5.10 ± 7.03	ns	6.55 ± 9.15	6.26 ± 9.19	ns
Average AI (*n*/hour) ± SD	1.97 ± 2.41	0.90 ± 0.72	ns	2.74 ± 6.30	0.75 ± 0.45	ns
Average HI (*n*/hour) ± SD	0.73 ± 0.94	0.36 ± 0.31	ns	0.78 ± 1.35	0.31 ± 0.25	ns
Average AHI (*n/*hour) ± SD	2.97 ± 4.46	1.26 ± 0.81	ns	3.52 ± 7.03	1.06 ± 0.53	ns
Central apnea index (*n*/hour) ± SD	0.66 ± 1.36	0.14 ± 0.18	ns	0.70 ± 1.59	0.11 ± 0.14	ns
Obstructive apnea index (*n/*hour) ± SD	1.12 ± 1.35	0.62 ± 0.57	ns	1.79 ± 5.76	0.54 ± 0.45	ns

SD, standard deviation; Avg., average; AI, apnea index; HI, hypopnea index; AHI, apnea-hypopnea index.

## Data Availability

The data presented in this study are available on request from the corresponding author.
